# Barriers and solutions in cross-sector care for metastatic prostate cancer patients in Germany: a qualitative study on radioligand therapy

**DOI:** 10.1186/s12913-025-13540-9

**Published:** 2025-10-02

**Authors:** Carolin Schenzle

**Affiliations:** Department Health and Sport Sciences, School of Medicine and Health, Technical University of Munich, Uptown Munich-Campus D Georg-Brauchle-Ring 60/62 D-80992, Munich, Germany

**Keywords:** Radioligand therapy, Prostate cancer, [177Lu]Lu-PSMA, [68 Ga]Ga-PSMA, PET/CT imaging, PSMA

## Abstract

**Background:**

In December 2022, Pluvicto^®^ (lutetium(177Lu)-vipivotide tetraxetan) received European Medicine Agency approval based on the Phase III VISION study as the first radioligand therapy (RLT) directed against prostate-specific membrane antigen (PSMA) and using Lutetium-177 (^177^Lu) for the treatment of metastatic prostate cancer. The objective of this study was to explore current barriers to the provision of ^177^Lu-PSMA RLT by drawing on the experiences of providers and to outline potential solutions.

**Methods:**

Eighteen physicians involved in the care of metastatic prostate cancer in the outpatient and inpatient settings from different regions of Germany participated in this semi-structured, qualitative expert interview study. Qualitative content analysis with inductive category formation was used to analyze the data.

**Results:**

Four major thematic categories were identified regarding barriers to care: (a) research-practice gap, (b) challenges to interprofessional collaboration, (c) resource constraints, and (d) unwarranted variation in care. Expert suggestions for resolving these barriers were grouped into the corresponding major thematic categories: (a) knowledge management, (b) integration of care, (c) capacity planning, and (d) facilitation of access.

**Conclusions:**

The delivery of ^177^Lu-PSMA RLT faces several obstacles, including challenges in collaboration, resource allocation, and reimbursement solutions. Minimizing bureaucratic hurdles in forming healthcare teams is essential to enhance interprofessional coordination. Resources should be evaluated and distributed based on local needs, and expanding reimbursement options for prior diagnostics is critical to ensure early treatment access. Overcoming these barriers and adapting health systems to novel therapies like RLT are essential steps toward improving cancer care and patient outcomes.

**Supplementary Information:**

The online version contains supplementary material available at 10.1186/s12913-025-13540-9.

## Background

Radioligand therapy (RLT) with Lutetium-177 (^177^Lu)-labeled prostate-specific membrane antigen (PSMA)-antagonists uses a personalized approach in which it harnesses PSMA on the surface of degenerated prostate cells, thereby enabling targeted radiation with sparing of surrounding tissue [1, 2]. In the VISION trial, a randomized multicenter open-label phase III study with 831 participants, the radioactive drug ^177^Lu vipivotide tetraxetan showed significantly prolonged progression-free survival (median 8.7 vs. 3.4 months) and overall survival (median 15.3 vs. 11.3 months) in patients with progressive metastatic castration-resistant prostate cancer (mCRPC) when combined with the standard therapy [3, 4]. Patients eligible for the study were required to have received at least one AR pathway inhibitor treatment, and one or two prior taxane-based chemotherapy regimens [4]. On December 9, 2022, the drug received approval from the European Medicines Agency for the patient population with mCRPC and is now marketed by Novartis under the name Pluvicto^®^ [5–9]. The approval increases the number of patients to be treated with RLT, a trend that is predicted to continue in the future as RLT is a rapidly evolving field and further clinical trials are underway to determine the applicability of Pluvicto^®^ in earlier disease stages [8, 10–12]. However, research shows that health care systems are often inadequately prepared for the integration of RLT [13]. As RLT with ^177^Lu-labeled PSMA antagonists is the first RLT with PSMA to be approved for patients with advanced prostate cancer (PC), careful planning is needed to ensure the integration of RLT into care systems and to guarantee patient access [8, 12]. The involvement of key professionals in the care process provides an important basis for the development of new forms of care [14]. Since the approval of Pluvicto^®^, no study has examined the current delivery and organizational challenges of care regarding the provision of RLT treatment for patients with metastatic prostate cancer (mPC) in Germany. Studies that have investigated the situation in Europe, the United States and the Netherlands provide indications for barriers, including poor understanding and skepticism against RLT among physicians, lack of personnel and centers offering the therapy, as well as the absence of clearly defined working processes. Besides focusing on other health care systems, these studies summarized barriers for different types of RLTs and different diseases [12, 15, 16]. In a computational study addressing the therapy bed capacity for ^177^Lu-PSMA RLT in Germany, Zippel et al. [17] predicted the number of therapy beds to be a bottleneck factor in the event of an approval. The question is to what extent barriers emanating from previous studies also apply to RLT in Germany and whether there are further barriers that have not yet been identified.

## Methods

This study intended to offer an informative foundation for ^177^Lu-PSMA RLT care planning by identifying barriers to care across the sectors of the German healthcare system and corresponding solution approaches with the help of physicians involved in the care of mPC. The research questions to be answered are as follows:

1. What current barriers to cross-sector care for mPC with ^177^Lu-PSMA RLT do physicians in Germany experience in their daily practice?
2. What solutions to the identified barriers do physicians propose based on their experiences in daily practice to improve patient care?

Semi-structured interviews with experts in the care of patients with mPC served as the data collection method. The development of the interview guide (Additional file 1) followed the process of operationalization by Kaiser [18], with the analysis dimensions adopting the understanding of cross-sectoral care by the German Managed Care Association [19] and Korzilius [20]. Maximum variation sampling as a method of purposive sampling was used to select the sample. The method was chosen to consider regional differences and the perspectives of the sectors and various specialist groups. As part of the sampling method, from North-South, East and West Germany, one doctor from the specialist groups of nuclear medicine, urology and oncology was invited for the outpatient sector and one doctor from the specialist groups of nuclear medicine and urology for the inpatient sector. The target sample size was consistent with Onwuegbuzie & Leech’s [21] guideline size of 15–20 respondents for semi-structured interview studies. The contacts used for this outreach were those available in the Customer Relationship Management System of Novartis. Contact was made by email, enclosing personalized invitation flyers with basic information about the project. To compensate for potential loss of earnings due to participation, physicians were offered compensation in the amount of 150–200 euros, depending on fair market value. The final sample consisted of 18 (Table 1) physicians. Interviews took place via Microsoft Teams between June 20 and September 11, 2023, each lasting approximately 40 minutes. All interviews were recorded and transcribed according to Mayring’s [22] transcription rules. After transcription, participants received their transcripts and had the opportunity to request corrections. The interview material underwent qualitative content analysis following Mayring’s [23] methodology, utilizing inductive category formation and the QCAmap. The units of analysis were specified as follows:

- Evaluation unit: the entire text material (18 transcripts)
- Context unit: the whole interview
- Coding unit: clear semantic elements

**Table 1:** Professional characteristics of the participants

Interview	Sector	Specialist group	Federal state	ASV^a^
1	Outpatient	Internal medicine and hematology and oncology	Hesse	Yes
2	Outpatient	Urology + med. TT	Saxony-Anhalt	No
3	Outpatient	Nuclear medicine	Hamburg	Yes
4	Outpatient	Urology + med. TT	Schleswig-Holstein	No
5	Outpatient	Urology + med. TT	Hamburg	Yes
6	Inpatient	Urology + med. TT	Hamburg	In process
7	Outpatient	Nuclear medicine	Bavaria	Yes
8	Outpatient	Urology	Bavaria	No
9	Outpatient	Nuclear medicine	Saxony	No
10	Outpatient	Urology + med. TT	Rhineland Palatinate	No
11	Inpatient	Nuclear medicine	Baden-Wurttemberg	Yes
12	Inpatient	Nuclear medicine	Mecklenburg-West Pomerania	No
13	Inpatient	Nuclear medicine	North Rhine-Westphalia	Yes
14	Inpatient	Urology + med. TT	Saxony-Anhalt	Yes
15	Inpatient	Nuclear medicine	North Rhine Westphalia	No
16	Inpatient	Nuclear medicine	Lower Saxony	In process
17	Inpatient + outpatient	Nuclear medicine	Hesse	Yes
18	Inpatient	Urology + med. TT	Bavaria	No

*ASV* outpatient specialist care; *Med. TT* additional training in medical tumor therapy. Two physicians were in the process of building an ASV team

^a^Membership in an ASV team for urological tumors

Selection criteria can be seen in the Additional file 2. A minimum of two appearances in different interviews was set as the threshold for categories relating to barriers.

## Results

**Fig. 1:**
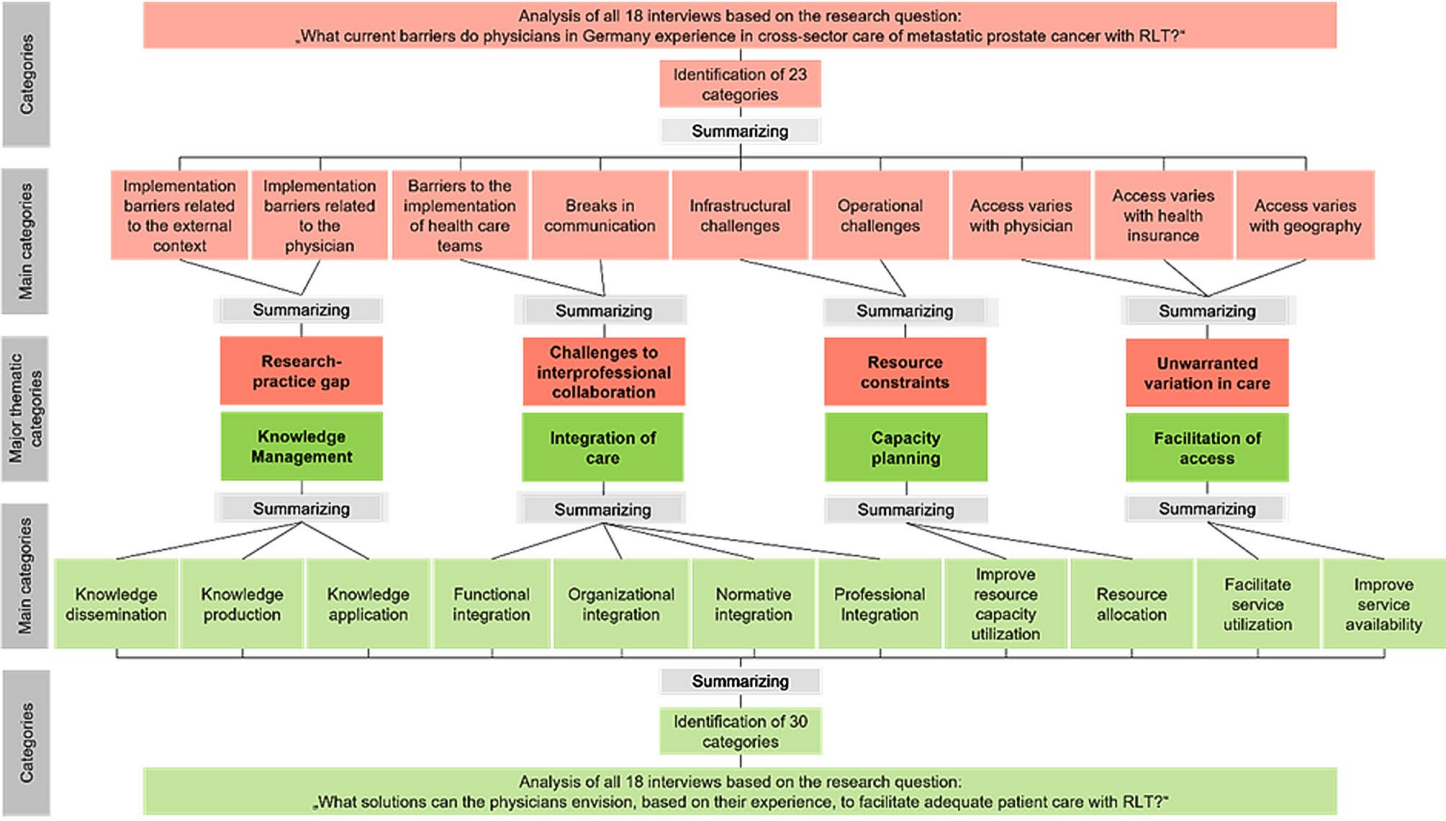
Category system of barriers and solutions in the care of radioligand therapy

The analysis of the interview material based on the first research question yielded 23 categories. These were combined into main categories and grouped into four major thematic categories: (a) research-practice gap, (b) challenges to interprofessional collaboration, (c) resource constraints, and (d) unwarranted variation in care. For the second research question, the same procedure resulted in 30 categories, which were summarized and grouped into four major thematic categories: (a) knowledge management, (b) integration of care, (c) capacity planning, and (d) facilitation of access. Figure 1 depicts all categories, with barriers shown in red and solutions in green. In accordance with Mayring’s [23] quality criterion of semantic validity, all major thematic categories were assigned reference-supported definitions, anchor examples, and encoding rules (Additional file 3). Barriers related to a gap between research and practice (100% of documents) and challenges with interprofessional collaboration (94% of documents) were noted most frequently. Full frequency details are provided in Additional file 4 for barrier categories and in Additional file 5 for solution categories.

### Research-practice gap

Among *implementation barriers related to the external context*, the limitations of the S3 guideline on prostate carcinoma were cited most frequently. The S3 guideline for prostate cancer is a German evidence-based clinical protocol that provides detailed recommendations for the prevention, diagnosis, treatment, and follow-up of prostate cancer, integrating scientific evidence, clinical expertise, and patient preferences to ensure standardized, high-quality care [24]. The interviewees described the guideline as outdated and the wording as too flexible [25]. Because of the perceived limited value, physicians reported following European or international guidelines instead [26, 27]. Regarding implementation barriers, physicians also reported hesitations in medical decision-making, particularly concerning the optimal timing of Pluvicto^®^ use and the management of patients ineligible for prior chemotherapy or experiencing disease progression during treatment. Criticisms were directed at the available study data, which were perceived as unrepresentative of routine clinical practice in Germany, with inclusion criteria and treatment pathways not accurately reflecting real-world scenarios. These uncertainties were further compounded by perceived financial risks related to interventions by the Medical Service (MD, Medizinischer Dienst) and ongoing negotiations for reimbursement of new diagnostic and treatment methods (NUB, Neue Untersuchungs- und Behandlungsmethoden) [28]. Uncertainties were perceived as a “stumbling block” (Interview 4 l. 101) to the use of RLT. Additional barriers to RLT implementation included regulatory interventions in therapy or diagnostic use that experts considered unjustified and obstructive to patient access. Interviewees particularly criticized the frequent rejection of cost claims for positron emission tomography/computed tomography (PET/CT) and the MDs disregard of medical recommendations when evaluating Pluvicto^®^ use. Disagreements with the MD often centered on the timing of therapy, as the MD relied on the S3 guideline for prostate carcinoma in effect at the time–which did not yet incorporate the new approval–while physicians referred to the Pluvicto^®^ label. The main category *Implementation barriers related to the physician* reveals that physicians can also be a barrier in implementing evidence into patient care. Analysis of the interviews suggested that some referring urologists may be unaware of the availability of ^177^Lu-PSMA RLT or reluctant to modify established clinical practices, thereby limiting patient access.

### Challenges to interprofessional collaboration

Among *barriers to the implementation of health care teams,* the most frequently cited were the bureaucratic hurdles of outpatient specialist care (ASV, ambulante spezialfachärztliche Versorgung). Physicians described the ASV as a “bureaucratic monster” (Interview 6, l. 211; Interview 9, l. 25), highlighting the substantial effort required to establish and maintain ASV teams. High bureaucratic hurdles were cited as a major deterrent to engaging in ASV, compounded by perceived limited medical and financial benefits. Practical challenges also arose with tumor boards, which are often held exclusively on-site; as travel time is not reimbursed, participation was described as a “waste of time” (Interview 3 l. 222) which one “cannot afford” (Interview 2 l. 90) in the office-based setting. These obstacles reportedly led to decisions against participation, with most mCRPC patients not being presented to the tumor board. Interprofessional rivalries, including concerns about patient poaching and preference for personal therapy choices, were reported to hinder collaboration. Skepticism about tumor boards among outpatient physicians–largely due to the perceived anonymity of clinical decision-making–further limited participation, with some seeing little added value and slower decision-making. Most *breaks in communication* occurred between outpatient and inpatient sectors, although shortcomings were also noted between outpatient practices.

### Resource constraints

Regarding *infrastructural challenges*, experts most frequently criticized long waiting times for PET/CT. These delays were attributed to a lack of available PET/CT equipment, with high investment costs for providing PSMA-PET/CT identified as a key obstacle. For Gallium-68 (^68^Ga) imaging, necessary investments were reported to include not only the substantial cost of a PET scanner but also the purchase of a gallium generator and compliance with regulatory requirements. Experts reported *operational challenges* with similar frequency to infrastructural challenges. They highlighted insufficient time for patient care, exacerbated by the fact that consultations with mPC patients - often elderly and facing emotional or cognitive burdens from their illness or prior treatments – are not reimbursable. Patient management was further complicated by limited software solutions, with medical reports still sent by fax and ongoing issues in telematics infrastructure. Personnel shortages were also noted, including nuclear medicine physicians and nurses, alongside increased need for administrative staff to handle the additional organizational workload associated with monitoring patients under Pluvicto^®^ administration.

### Unwarranted variation in care

This major thematic category captures deviations in patient care that cannot be attributed to variations among patients themselves. The main category of *access varies with physician* illustrates that the referring physician has a decisive influence on the patient’s course of treatment. Both the physician’s ASV membership status and level of knowledge were reported to affect access. Furthermore, *access varies with health insurance*, indicating that the type of coverage can affect access to both PET/CT and RLT. At the time, this was mainly due to the absence of reimbursement options for PET/CT scans outside ASV, missing uniform value scale (EBM, Einheitlicher Bewertungsmaßstab) codes and ongoing negotiations regarding NUB fee structures with health insurers. Reimbursement for private patients was generally described as unproblematic. Geographic factors further influenced access: *Access varies with geography* and the patient’s residence, with long travel distances in rural areas reported to discourage patients and affect urologists’ referral decisions, especially when local alternatives such as chemotherapy were available. 

### Knowledge management

This major thematic category summarizes ideas for bridging the identified gap between research and practice. One component, knowledge dissemination, includes educating physicians via congresses or apps, timely guideline updates, interprofessional networking events, and training regulatory staff on relevant 177Lu-PSMA-RLT regulations. Knowledge production involves expanding studies and establishing a registry to collect real-world evidence. Finally, knowledge application highlights physicians’ desire for greater professional autonomy in making medical decisions regarding 177Lu-PSMA-RLT or PET/CT.

### Integration of care

This major thematic category contains ideas on different ways to integrate providers and improve collaboration. Suggestions for *functional integration* included virtual tumor boards, incentives for health care team participation, and software solutions for interprofessional communication. Further approaches were virtual meetings for consultation between physicians, digitization among agencies to streamline bureaucratic processes, exclusion from ASV in case of non-participation in the tumor board and softening inclusion criteria for ASV such as the distance criterion of a maximum of 30 min to the location of the team lead, and obligation to provide a second medical opinion. Other proposed solutions for integrating care focused on service coordination and were grouped under the main category of *organizational integration*. Suggestions included pooling PET/CT centers (Interview 9), combining interdisciplinary therapy stations within clinics to overcome rigid specialty boundaries (Interview 12) and consolidating ASV teams to establish one team per tumor entity and state, lowering participation barriers (Interview 9). Another proposal involved designating a permanent clinic contact person for referrals to streamline and accelerate patient presentation for RLT (Interview 1). *Normative integration* focused on raising awareness of the need for collaboration within modern cancer care. *Professional integration* could be achieved by aligning on defined patient flows between sectors and mitigating competitive fears through collegial trust and fair agreements.

### Capacity planning

To address resource scarcity, one approach was to *improve resource capacity utilization* by using SPECT/CT instead of PET/CT for faster coverage and implementing software for patient management. R*esource allocation* measures included the introduction of oncological navigators. Introducing a billing code for patient consultations and financial protection from the pharmaceutical company in case of therapy-day cancellations was also suggested to integrate counseling into routine practice and reduce financial risk associated with Pluvicto^®^ use.

### Facilitation of access

Easing access to ^177^Lu-PSMA-RLT and PET/CT encapsulates the approach to *facilitate service utilization*, among which the need for the introduction of an EBM code was mentioned most frequently. Including nuclear medicine specialists in the ASV core team was suggested to further promote the spread of ASV teams. The physician’s role was emphasized as critical in encouraging patients to adhere to necessary therapy, despite the potential challenges posed by long-distance travel. A further approach was to *improve service availability*, for example by fully utilizing existing local supply structures. 

## Discussion

This study is the first qualitative investigation of ^177^Lu-PSMA-RLT care for prostate cancer patients in Germany, identifying barriers across knowledge, stakeholder integration, access, and capacity planning. It became evident how ambiguities in the physician’s environment, such as disagreements with health insurers on cost issues due to divergences in guidelines, can have a delaying effect on the implementation of ^177^Lu-PSMA-RLT in practice and enforce professional uncertainty in its application. After the completion of this study, on October 1, 2023, ^68^Ga-PSMA-PET; PET/CT became reimbursable under the EBM for determining the indication for ^177^Lu-PSMA-RLT [29]. Fluorine-18(^18^F)-PSMA-PET; PET/CT can still only be reimbursed within the framework of ASV. The same applies to ^68^Ga- or ^18^F-PSMA-PET; PET/CT for further indications such as propagation diagnostics in high-risk PC or diagnostics in the event of PSA recurrence in localized PC [30]. After the approval of Pluvicto^®^, the treatment was still classified as a treatment attempt within the valid version (6.2) of the guideline [25]. It was only in May 2024 that a new version (7.0) of the guideline was published, wherein Pluvicto^®^ was included as a standard therapy, which can be administered in accordance with its label after the necessary prior treatments [31]. Decreasing the intervals between updates on the S3 guideline for prostate carcinoma is a pivotal initial stride towards ensuring improved guidance for physicians, and instilling confidence in the utilization of novel therapies. The findings of the current study underscore the pressing need for these reforms to secure patients’ access to ^177^Lu-PSMA RLT. While certain findings of this study parallel those from research conducted in other countries or focused on different diseases, new insights were uncovered specifically pertaining to the usage of ^177^Lu-PSMA RLT in Germany. Consistent with existing literature, experts recounted instances where a lack of familiarity with ^177^Lu-PSMA RLT was linked to reservations and subsequent delays in its application [15]. Contrary to previous research that highlighted physicians’ safety concerns as a deterrent, participants in the present study expressed confidence in the ^177^Lu-PSMA RLT from a medical standpoint. The approval granted in the meantime may have increased the medical community’s trust in the therapy. However, financial concerns persist despite this medical confidence. These external factors have not been reported for ^177^Lu-PSMA RLT since its approval and include a lack of assurance from health insurance providers concerning NUB negotiations or PET/CT reimbursement and actions taken by authoritative agencies regarding optimal therapy timing. Debates involving reimbursement were previously reported in the context of the therapy prior to its approval [2, 32]. Complexity of regulations in the health care system, leading to a prevailing sentiment among health care professionals that they dedicate more time to formalities than to patient care, has already been documented within the context of the American health care system [33]. However, according to the experts, regulatory disagreements not only lead to laborious paperwork but also delay and obstruct patient care. Therefore, MD discussions may hinder the implementation of this new therapy. While guidelines offer valuable direction for patient care, they cannot account for every individual case, highlighting the importance of consensus decisions by interdisciplinary tumor boards [2, 34]. Similarly, experts in this study expressed a desire for greater autonomy in medical decision-making within an interdisciplinary team. The study identified obstacles that hindered the formation of interdisciplinary teams and limited physician participation in existing healthcare teams. Experts reported dissatisfaction with tumor board implementation, a finding consistent with prior research but new for mCRPC management in urological boards [35–38]. A German survey of 612 physicians (May 2021–Nov 2022) found that 33% felt patients’ wishes were inadequately considered [38]. Further studies reported this issue leading to inappropriate treatment recommendations [35, 36, 39–43]. Absence of a physician with personal contact with the patient and incompleteness of information for decision-making were reported [38]. The primary issue revealed in this study is the apparent challenge for office-based urologists in the practicability of tumor boards. Consistent with this finding, survey data show that 51% of tumor boards in Germany in 2022 were offered exclusively in presence [38]. Concerns about the time required for preparation and participation and missing financial compensation were highlighted in the literature [37, 44, 45]. Another barrier to participation, identified by experts, centers on the perceived benefits of tumor boards. Various studies in the literature yield heterogeneous results pertaining to the benefits of such boards [39, 41, 46–51]. Researchers attribute the heterogeneity to a high degree of variation in the way tumor boards are practiced. Differences were found in the format (presence or on-site), number of participants and presented patient, and duration [38, 52]. Previous studies suggest that nurse-patient involvement is a way to increase patient-centeredness in the tumor board. However, because there is evidence of limited feasibility of this approach in routine care, the proposal is limited to selected cancer patients [53–56]. In the current study, experts suggested alternative methods to increase mCRPC patient representation at tumor board sessions, including the broadening scope of virtual tumor boards. Digital participation has been suggested in the literature as a feasible way to overcome geographical barriers to tumor board attendance [38, 57]. Regarding the identified doubts about the benefits of tumor boards, analyses in England show that adherence of health care teams was higher in cancer entities with early guideline establishment for multidisciplinary collaboration [58]. Taylor et al. [59] assume an initial lack of readiness in changing established work practices, which is why team involvement improves as health care systems become more ingrained [59]. Accordingly, team participation may continue to improve as the interdisciplinary approach is further consolidated in the coming years. Existing research indicates that with an increasing prevalence of tumor boards and the associated routine collaboration, improvement in cooperation between office-based and clinic-based physicians and an increase in trust and understanding can be expected [52, 60]. The interviews revealed barriers to the broader adoption of the ASV concept for urologic tumors, with high bureaucracy and limited therapeutic or financial incentives cited as major obstacles. Consistent with a survey by Dengler et al. [61] nonparticipants reported high bureaucratic burden (62.7%) and lack of strategic (32.2%) and financial benefit (31.6%) as key deterrents. Motivating factors included overcoming sector barriers, interdisciplinary care, collaboration with other specialists, and billable services. Regarding collaboration, differences in the use of digital communication channels between outpatient and inpatient sectors were noted. Outpatient physicians reported limited digitization in hospitals. An IGES Institute evaluation [62] from June 2022 found a 19% gap in Nutzung von Kommunikation im Medizinwesen (KIM, communication in medicine between sectors) with usage rates low in both (10% in hospitals vs. 29% in office-based practices). Interviewees’ dissatisfaction with the telematics infrastructure aligns with these findings, as 42% of hospitals and 74% of practices reported frequent connection issues. The study indicated that differing use of digital communication tools between facilities may cause intersectoral communication breaks and delays, highlighting the need to promote adoption and provide support across both sectors. In addition to missing digitization, the present study identified rivalries among physicians as a challenge for collaboration and the establishment of healthcare teams. Related to this, a German study concerning the concept of tumor boards points to tensions between physicians involved from the outpatient and inpatient sectors that can affect treatment decisions [52]. However, the fact that these rivalries may also represent a barrier to participation in ASV teams has not yet been documented. The experts’ reports of staff shortages and limited time capacity with the patient and impacts in terms of lacking patient guidance are consistent with studies from the UK, Canada, and Australia on cancer patients receiving precision medicine, radiotherapy or different types of RLTs [15, 63, 64]. To date, there have been no studies in Germany that, like the present interview study, reported additional organizational workload and lack of staff capacity associated with ^177^Lu-PSMA therapy. The reported experiences of lengthy waiting times for PSMA PET/CT are novel within the literature, indicating an urgent call to action given the advanced stage of metastatic Prostate Cancer (mPC) and the associated time pressure. In the current study, a proposal for overcoming deficits in patient navigation and the counselling burden associated with elderly oncology patients was seen in the introduction of oncology pilots. In the United States, patient navigation programs have been standard in the National Accreditation Program for Breast Centres since 2009, involving trained nurses, social workers, and lay personnel [65]. Interventions indicate that navigation of PC patients results in fewer missed clinic appointments and earlier initiation of diagnosis and treatment [65–67]. Further research is needed to evaluate whether oncology navigators in Germany could improve survival, quality of life, and cost-effectiveness for prostate cancer patients, and to establish criteria for identifying patients with special needs. To reduce diagnostic waiting times, experts proposed using SPECT/CT as an alternative to PET/CT. Evidence suggests that PSMA SPECT/CT may substitute for PSMA PET/CT in advanced mPC [68]. Given its greater availability and cost-effectiveness, SPECT could enable faster and broader patient access across many centres [68–70]. During the expert interviews, a variation in the care of patients with mCRPC became apparent that was not attributable to differences in patients’ wants, needs, or disease stage, but rather to current issues in reimbursement, geographic gaps in care, and lack of awareness on the part of referring physicians. In particular, the experts interviewed highlighted the limited distribution of ASV, as at the time outpatient PET/CT services under SHI could only be reimbursed through this pathway. In line with Dengler et al. [61], the experts also described the distance criterion of 30 minutes from the team leadership as a bureaucratic obstacle that further complicates team building, particularly in rural areas, and should be softened or removed. Overall, multiple areas were identified that need to be improved in the future to provide patients with the best possible care with this innovative therapy. It should be emphasized that the results described are not intended to deliver a comprehensive overview of all barriers associated with ^177^Lu-PSMA RLT care. Rather, this study should be seen as a significant first step in deciphering the complex care of mCRPC patients in Germany. It attempts to identify potential starting points that need to be further investigated and refined in subsequent studies. This study has strengths and limitations that should be considered when interpreting the findings and designing future research. Although the honorarium was deliberately kept low to compensate for time and effort rather than serve as a financial incentive, participants–recruited via Novartis’ CRM system–may have been somewhat more positively inclined toward RLT than the target population. To enhance robustness and minimize potential selection bias, a threshold of two interviews was set for category inclusion, ensuring that reported themes reflect recurring rather than singular perspectives. A major strength of the sample lies in its targeted design, including stakeholders from both sectors and various specialties, while ensuring balanced representation across regions in Germany. This reinforces the sampling validity as a quality criterion of qualitative content analysis. As interviewees highlighted the substantial organizational demands of implementing RLT, future studies should also involve patients and their relatives, nursing staff, and administrative personnel to provide a more holistic view of both organizational burden and treatment experience. Furthermore, objectivity represents a critical point in qualitative content analysis, as it relies on the interpretation of text. Complete objectivity cannot be achieved as the researcher’s prior knowledge–including insights from the literature review used to develop the interview guide–shaped the analysis. However, the rule-governed procedure of qualitative content analysis, combined with intracoder agreement tests, helped to enhance stability and reproducibility. Using a semi-structured guide allowed comprehension problems to be addressed during the interviews, and physicians were contacted afterwards when passages were unclear. An advantage of content analysis is its ability to quantify frequencies when naming categories, providing an indication of which barriers are most pressing and should be prioritized when designing solutions. Additional strengths and weaknesses are found in the selection of interviews as the method of data collection. Face-to-face interviews are subject to the risk of social desirability bias [71]. To mitigate such biases, experts were afforded the opportunity to propose amendments to their post-interview statements in the transcripts, complemented by the assurance of their anonymity within this paper [72]. In summary, this study provides novel insights into the care of patients receiving ^177^Lu-PSMA-RLT in Germany that have not been previously reported in European or US contexts. Specifically, it highlights organizational challenges, including increased staff workload, intersectoral communication breaks, and limited digital infrastructure, as well as barriers related to ASV team formation and tumor board participation. Furthermore, it identifies variations in patient access to PET/CT and RLT driven by reimbursement structures, geography, and referring physician awareness. A key limitation of this study is that only physicians’ perspectives were included; input from patients, caregivers, and nursing staff is needed in future research to obtain a more comprehensive understanding of both the organizational burden and the patient treatment experience. 

## Conclusions

This qualitative study highlights the hurdles hindering the delivery of ^177^Lu-PSMA RLT, including challenges related to collaboration, resource constraints, and reimbursement solutions. Minimizing bureaucracy to support better interprofessional coordination and optimizing resource allocation based on local needs are essential. Expanding reimbursement options for prior diagnostics is pivotal to enabling early access to treatment. Qualitative research is an important component to better understand weaknesses in current approaches to care and develop pathways to improve health care performance and patient-centeredness based on those weaknesses. Further in-depth and continuous research is paramount in striving to provide patients with the best possible care. The ability of health systems to adapt swiftly and effectively to novel therapies like RLT will play a critical role in shaping the future of cancer care and improving patient outcomes worldwide.

## Abbreviations

**Table:** 

Abbreviation	German	English
^18^F		Fluorine-18
^68^Ga		Gallium-68
^177^Lu		Lutetium-177
ASV	ambulante spezialfachärztliche Versorgung	Outpatient specialist care
CT		computed tomography
EBM	Einheitlicher Bewertungsmaßstab	Uniform value scale
mCRPC		Metastatic castration-resistant prostate cancer
MD	Medizinischer Dienst	Medical service
mPC		Metastatic prostate cancer
MVZ	Medizinisches Versorgungszentrum	Medical care center
NUB	Neue Untersuchungs- und Behandlungsmethoden	New examination and treatment methods
PC		Prostate cancer
PET/CT		Positron emission tomography/computed tomography
PSMA		Prostate-specific membrane antigen
RLT		Radioligand therapy
SPECT/CT		Single photon emission computed tomography/computed tomography

## Electronic supplementary material

Below is the link to the electronic supplementary material.

Supplementary Material 1

Supplementary Material 2

Supplementary Material 3

Supplementary Material 4

Supplementary Material 5

## Data Availability

Interview transcripts generated and analyzed during the current study are not publicly available due to the protection of the participant’s privacy. Additional, fully anonymized, and aggregated data is however available from the author upon reasonable request.

## Declarations

### Authors’ information

This thesis was written as part of a master’s thesis for the M.Sc. Health Science degree at the Technical University of Munich. The work was supervised by the Chair of Health Economics at the Technical University of Munich and Novartis Radiopharmaceuticals GmbH.
