# Supplementary material for: Barriers and solutions in cross-sector care for metastatic prostate cancer patients in Germany: a qualitative study on radioligand therapy
Source: BMC Health Serv Res. 2025 Oct 2;25:1281. doi: 10.1186/s12913-025-13540-9 (PMC12490126; doi:10.1186/s12913-025-13540-9)
Supplement: Supplementary file 1 — Supplementary Material 1 [file 12913_2025_13540_MOESM1_ESM.pdf]

**Additional file 1**. Interview Guide

<u>Introduction</u>
Welcome and introduction
Aim of the interview

<u>Background</u>
Please describe your role as [job description] in the care of mPC
Can you describe how the care of patients with mPC is organized in your institution?

| **Continuous treatment process** | |
|---|---|
| **Barriers** | |
| In your experience, are there areas where there can be interruptions or delays in the course of treatment when using an RLT? | |
| *Further Questions* | *How smooth is the transfer of the patient group between facilities?*<br>*In your opinion, do patients have adequate access to RLT?*<br>*How do you assess the provision of resources for RLT and its diagnostics (raw materials, equipment, staff, buildings) at the facilities?*<br>*How are the local care structures (close to home) organized?* |
| **Solutions** | |
| Can you think of specific measures that would facilitate the seamless integration of RLT into the treatment process if indicated? | |
| *Further Questions* | *Could digital solutions increase efficiency in the treatment process? What could they look like?* |

| **Patient orientation** | |
|---|---|
| **Barriers** | |
| To what extent can patients' needs and wishes for RLT be taken into account? | |
| *Further Questions* | *In your opinion, how informed are patients about the treatment options available to them?*<br>*How important is the patients' level of knowledge in the treatment of mPC?* |
| **Solutions** | |
| What requirements must be met to ensure that RLT can be based on the patient's wishes? | |

| **Shared responsibility** | |
|---|---|
| **Barriers** | |
| With the increasing number of treatment options, ethical, legal, economic, and social issues must be considered. What challenges are there when deciding on an RLT therapy? | |
| *Further Questions* | *What role do economic/legal factors play in the treatment decision for/against RLT?*<br>*What possibilities does the old supply situation in mPC offer for the doctor-patient discussion?* |
| **Solutions** | |
| Which measures create optimal conditions for a correct treatment decision and support the doctor? (remuneration, legal, etc.) | |

| **Consideration of guidelines** | |
|---|---|
| **Barriers**<br>In your opinion, to what extent is the treatment of metastatic prostate cancer with RLT based on the S3 guideline on prostate cancer? | |
| *Further Questions* | *How satisfied are you with the guideline?*<br>*In your opinion, what role does the guideline play in the implementation of medical progress?*<br>*How consistent are the procedures for the treatment of metastatic prostate cancer among colleagues?* |
| **Solutions**<br>How should the S3 guideline on prostate cancer be designed to optimally support physicians in the provision of RLT? | |

| **Coordination, communication, cooperation** | |
|---|---|
| **Barriers**<br>Are responsibilities and accountabilities for the care of metastatic prostate cancer clearly defined across sectors? | |
| *Further Questions* | *How does collaboration between the sectors work (from referral to diagnostics to RLT)?*<br>*How does the cooperation within your sector work?*<br>*How does the coordination and transfer of information between the treating physicians of patients with mPC work?* |
| **Solutions**<br>What measures can facilitate cross-sectoral cooperation between the actors involved? | |

| **Optimal allocation** | |
|---|---|
| **Barriers**<br>How are patients with an indication for RLT guided through the healthcare system to the appropriate medical profession/facility? | |
| *Further Questions* | *How pronounced is the role of multidisciplinary teams in this management?*<br>*How is network work among physicians remunerated?*<br>*What is your view of the ASV concept for urological tumours? Why are you a member/not a member?* |
| **Solutions**<br>What measures can you think of to increase participation in multidisciplinary teams? | |
