# Supplementary material for: Barriers and solutions in cross-sector care for metastatic prostate cancer patients in Germany: a qualitative study on radioligand therapy
Source: BMC Health Serv Res. 2025 Oct 2;25:1281. doi: 10.1186/s12913-025-13540-9 (PMC12490126; doi:10.1186/s12913-025-13540-9)
Supplement: Supplementary file 2 — Supplementary Material 2 [file 12913_2025_13540_MOESM2_ESM.pdf]

Additional file 2. Selection Criteria for Barriers and Solutions

Criterion	Barriers	Solutions
Category definition	Factors that prevent or limit prostate cancer patients from accessing RLT and/or achieving their best possible health. These factors can be shaped by a wide range of forces, including economic, political and social forces. (45,46).	Problem solutions that physicians draw on the one hand from information from the environment and on the other hand from their previous experiences and prior knowledge in order to construct internal exploratory models of how a problem could be mastered (47,48).
Level of abstraction	Negative factors that may be specific to the physician or general in formulation and affecting patient care in general but in principle applicable also to other professionals involved in the care of the patient group. They must neither be past nor expected in the future but must be currently and personally experienced and not formulated hypothetically.	Solutions to previously identified problems may be specific to the respective physician and affecting patient care in general but must be applicable in principle also to the problems of other professionals involved in the care of the patient group and to the care situation of ^{177}Lu-PSMA RLT. Solution approaches can be based on conceptions or already be implemented in the sense of a showcase function. To have practical relevance, solution approaches further must incorporate a concrete example.
