# Supplementary material for: Barriers and solutions in cross-sector care for metastatic prostate cancer patients in Germany: a qualitative study on radioligand therapy
Source: BMC Health Serv Res. 2025 Oct 2;25:1281. doi: 10.1186/s12913-025-13540-9 (PMC12490126; doi:10.1186/s12913-025-13540-9)
Supplement: Supplementary file 3 — Supplementary Material 3 [file 12913_2025_13540_MOESM3_ESM.pdf]

Additional file 3. Main Categories and Corresponding Definitions, Anchor Samples and Encoding Rules

Category	Definition	Anchor sample	Encoding rule
Research-practice gap	Refers to the time lag between scientific findings and their translation into clinical practice occurring if new insights cannot be promptly translated into patient care and corresponding health improvements (22–26). Elements of evidence-based medicine include the production of evidence through research that must be applicable to practice and the production and dissemination of evidence-based guidelines to support clinicians (25,27,28). Factors that hinder the implementation of evidence into practice may be related to the external context, the organization, the professionals, and the innovation itself can (29).	"[...] so that the main question now is actually when you should use it. How early or how late. The question is not easy to answer, the study situation is still in process, one must say. That is certainly a stumbling block, or not a stumbling block, but an uncertainty." (Interview 4, l. 99ff.)	Any system-level or provider-level factor that slows, complicates, or prevents the implementation of the therapy as an innovation. Includes the entire life cycle.
Challenges to interprofessional collaboration	Interprofessional collaboration is a form of cooperation between at least two healthcare professionals from different backgrounds that focuses on the joint achievement of a group goal (in this case, optimal patient care) and includes aspects of communication, coordination, and cooperation (30,31). A successful collaboration is characterized by a collegial relationship, where complementary competencies and skills of the participants merge to enable the achievement of the group goal, but implementation can be challenging (31,32). Barriers to successful collaboration may include interprofessional rivalries, inadequate reimbursement policies, complexity of the system, emphasis on rapid decision making, or professionals that are not convinced of the benefits of collaboration for patients (31,33).	"[...] Such a tumor board costs a lot of time and yields exactly zero euros. That is also a reason why we do not force ourselves to be there. If I write new findings during this time, it is more lucrative for my employer." (Interview 3, l. 199ff.)	Communication problems must be perceived as such by the professional, e.g., reports about a lack of digitalization are only incorporated, if experienced as a barrier that delays, impedes, or prevents optimal care
Resource constraints	Medical resource scarcity describes limited resources relative to need and can lead to competition between patients (34). Health resources reflect investments into the health care system, encompassing investments in personnel, equipment, and infrastructure needed to provide RLT (35). Resource constraints may impede or limit the adoption of RLT into clinical practice (36).	"[...] then he either has his wife with him, who makes sure that her husband is treated well, that he keeps his appointments, that he follows the doctors' advice and does this and that now. Or he's on his own and then, unfortunately, he falls over the backside far too often because the urologist who takes care of him doesn't have the time and the capacity to take care of it, to motivate the patient to really stick to everything that's been recommended." (Interview 3, l. 233-238)	Must have a relevant impact (direct or indirect) on provision of RLT

Unwarranted variation in care	Differences in care that are based on factors such as payment method, geography, or system inclinations rather than differences among patients such differences in patient illness or patient preferences (37,38).	"[...] it is not mapped at all in the EBM, that is, the catalog from which one can take the remuneration, which means that every patient, if he is not a private patient or says, I'll pay it myself now, is ultimately forced to either hope for a good will of the nuclear physician, who sits down and makes an application for cost coverage for the poor patient at the health insurance [...].This means that we submit an application for cost coverage for patients with statutory health insurance, and I would say that 80 percent are rejected." (Interview 9, l. 14-21)	Factors that may delay or prevent care for certain patients, the existence of differences must be mentioned explicitly
Knowledge Management	Umbrella term including the production and dissemination of knowledge to ensure its effective and efficient use (39,40). Dissemination of knowledge is a planned and active process to increase the level of knowledge adoption, for instance by the establishments of networking initiatives and the implementation of guidelines (40). The ability of applying the obtained knowledge in the decision-making processes of patient care is denoted as professional autonomy (41).	"[...] the decisive factor for this new product will be that the physicians are trained, that they are educated, that this option is available and when it is ready for use with which patients. I think Novartis, as they probably already know, has to push the training. Present it at the congresses, then train the urologists locally. We know that approval is expected, and it will then also be recommended in the tumor board. It then has to be implemented in everyday life." (Interview 1, l. 97-100)	Description of a way of becoming active
Integration of care	Integrated care describes the effort to connect the healthcare system and involves system, organizational, professional, clinical, normative, and functional integration. Functional integration achieves connectivity by support functions like financial, information and management systems while normative integration achieves connectivity by developing a common mission and common values. Thereby, both contribute to professional (partnerships between professionals), organization (service coordination) and system integration (alignment of policies within a system) (42).	"[...] that portals are created where these local networks are mapped. [...] everything is still done by hand, that you call there and present the patient. If there is then the health file and there are platforms created that these networks are supported, can communicate with each other, the patient file all results, pathology report, laboratory, everything is stored there [...]." (Interview 1, l. 314-319)	Corresponds to one of the different forms of integration
Capacity Planning	Resource planning strategies for healthcare resources include capacity extension (policy makers providing more resources), resource relocation (sharing of resources between facilities), as well as demand redistribution (redistribution of patients between facilities) (43).	"I don't know if it's possible to pool something like that, so that everyone enters their free dates somewhere and says, here, we can do this and that now. Maybe there is an option, if the University of Aachen has something free, then they say, we can do the next two weeks, we are standing with a fusion biopsy or with the ligand therapy, we would have a place free or if someone drops out, cancer patients drop out sometimes, that you then say, come here, I need someone to follow. That might be an option, a superordinate network where you can say that there might be places here and there where you could go." (Interview 10, l. 210-216)	Corresponds to one of the resource planning strategies

Facilitation of access	Facilitation of access is about helping people to command appropriate health care resources so that they can achieve their best possible health. The concept of access entails, first, the availability of an adequate supply of services (having access) and, second, the possibility to utilize these services (gaining access) which requires the removal of financial, organizational, and personal barriers (44).	"It would be desirable to make this analogous to the FDG-PET examinations as they are reimbursed for lung carcinoma, for example, which is the EBM number. There is then a fixed flat-rate item with which the examination is reimbursed plus another flat-rate for material costs. This is now cost-covering for FDG-PET [...]. And such a flat rate must be designed in such a way that the tracer costs are really covered [...]." (Interview 3, l. 104-109)	Concrete example, either improves service availability or facilitates utilization by reducing financial, organizational, or personal barriers

RLT radioligand therapy
