# Supplementary material for: Barriers and solutions in cross-sector care for metastatic prostate cancer patients in Germany: a qualitative study on radioligand therapy
Source: BMC Health Serv Res. 2025 Oct 2;25:1281. doi: 10.1186/s12913-025-13540-9 (PMC12490126; doi:10.1186/s12913-025-13540-9)
Supplement: Supplementary file 4 — Supplementary Material 4 [file 12913_2025_13540_MOESM4_ESM.pdf]

Additional file 4. Frequency of Occurrences of Barrier Categories

Category	Absolute count	% of SUM	N of documents	% of documents
Research-practice gap	25	22	18	100
Implementation barriers related to the external context	20	17	16	88
Limitations of the S3 guideline on prostate carcinoma	10	8	19	55
Professional uncertainty	5	4	5	27
Regulatory disagreements	5	4	5	27
Implementation barriers related to the physician	5	4	5	27
Lack of innovative behavior on the part of referring physicians	5	4	5	27
Challenges to interprofessional collaboration	44	38	17	94
Barriers to the implementation of health care teams	37	32	16	88
High bureaucratic hurdles regarding the ASV	12	10	12	66
Lack of benefits within the ASV framework	6	5	6	33
Practicability issues of tumor boards in the outpatient setting	6	5	6	33
Interprofessional rivalries	6	5	6	33
Skepticism about the competence of tumor boards among outpatient physicians	4	3	4	22
No perceived added value of tumor boards for decision-making	3	2	3	16
Breaks in communication	7	6	7	38
Intersectoral communication breaks	5	4	5	27
Communication breaks within the outpatient sector	2	1	2	11
Resource constraints	23	20	12	66
Infrastructural challenges	12	10	8	44
Waiting times for PET/CT	6	5	6	33
Lack of medical equipment	3	2	3	16
Costly investment barriers	3	2	3	16
Operational challenges	11	9	8	44
Insufficient time for patient services	4	3	4	22
Lack of software solutions	4	3	4	22
Personnel challenges	3	2	3	16
Unwarranted variation in care	21	18	12	66
Access varies with physician	8	7	6	33
Access to PET/CT varies with ASV-membership status	6	5	6	33
Access varies with awareness of the referring physician	2	1	2	11
Access varies with health insurance	8	7	6	33
Access to PET/CT varies with health insurance	6	5	6	33
Access to therapy varies with health insurance	2	1	2	11
Access varies with geography	5	4	5	27
Access to therapy varies with local supply structure	5	4	5	27

Major thematic categories are marked in dark grey, main categories in lighter grey, and normal categories are depicted without any color. The column *% of SUM* refers to the share of the sum (113 counts) of all counts in all categories. Categories are ordered according to their frequencies from high to low.
