# Supplementary material for: Barriers and solutions in cross-sector care for metastatic prostate cancer patients in Germany: a qualitative study on radioligand therapy
Source: BMC Health Serv Res. 2025 Oct 2;25:1281. doi: 10.1186/s12913-025-13540-9 (PMC12490126; doi:10.1186/s12913-025-13540-9)
Supplement: Supplementary file 5 — Supplementary Material 5 [file 12913_2025_13540_MOESM5_ESM.pdf]

Additional file 5. Frequency of Occurrences of Solution Categories

Category	Absolute count	% of SUM	N of documents	% of documents
Integration of care	24	34	13	72
Functional integration	19	27	11	61
Virtual tumor boards	5	7	4	22
Incentives for health care team participation	4	5	4	22
Software solutions for interprofessional communication	5	7	4	22
Virtual meetings for consultation between physicians	1	1	1	5
Digitization among agencies	1	1	1	5
Exclusion from ASV in case of non-participation in the tumor board	1	1	1	5
Softening inclusion criteria for ASV	1	1	1	5
Obligation to provide a second medical opinion	1	1	1	5
Organizational integration	4	5	3	16
Pooling of PET/CT centers	1	1	1	5
Interdisciplinary therapy stations	1	1	1	5
Pooling of ASV-teams	1	1	1	5
Contact person in the clinic for the referral	1	1	1	5
Normative integration	1	1	1	5
Create awareness among physicians for the need to collaborate	1	1	1	5
Professional integration	1	1	1	5
Agreement on rules for patient flow between sectors	1	1	1	5
Knowledge management	23	32	11	61
Knowledge dissemination	15	21	9	50
Education of physicians	6	8	5	27
Timely updating of guidelines	4	5	3	16
Organization of events to build interprofessional networks	3	4	2	11
Training of the regulatory staff	2	2	2	11
Knowledge production	4	5	2	11
Expansion of the study situation	4	5	2	11
Knowledge application	4	5	3	16
Professional autonomy	4	5	3	16
Capacity planning	9	12	7	38
Improve resource capacity utilization	5	7	5	27
Utilization of SPECT/CT instead of PET/CT	2	2	2	11
Software solutions for patient management	2	2	2	11
Capacity determination for resource planning	1	1	1	5
Resource allocation	4	5	4	22
Oncological navigators	2	2	2	11
Billing code for the patient consultation	1	1	1	5
Financial protection in case of cancellation on the therapy day	1	1	1	5
Facilitation of access	13	18	6	33
Facilitate service utilization	12	17	6	33
Full-coverage reimbursement of PET/CT outside the ASV	7	10	4	22

Inclusion of nuclear medicine specialists in the ASV core team	2	2	2	11
Motivation through patient education	3	4	1	5
Improve service availability	1	1	1	5
Fully utilize available local supply structures	1	1	1	5

Major thematic categories are marked in dark grey, main categories in lighter grey, and normal categories are depicted without any color. The column *% of SUM* refers to the share of the sum (70 counts) of all counts in all categories. Categories are ordered according to their frequencies from high to low.
